# Current and Emerging Diagnostic, Prognostic, and Predictive Biomarkers in Head and Neck Cancer

**DOI:** 10.3390/biomedicines12020415

**Published:** 2024-02-10

**Authors:** Hänel W. Eberly, Bao Y. Sciscent, F. Jeffrey Lorenz, Eleni M. Rettig, Neerav Goyal

**Affiliations:** 1 Department of Otolaryngology Head and Neck Surgery, Penn State Milton S. Hershey Medical Center, Penn State College of Medicine, Hershey, PA 17033, USA; hwatkins@pennstatehealth.psu.edu (H.W.E.); florenz@pennstatehealth.psu.edu (F.J.L.); 2 Department of Otolaryngology Head and Neck Surgery, Brigham and Women’s Hospital, Dana Farber Cancer Institute, Harvard Medical School, Boston, MA 02108, USA

**Keywords:** biomarkers, head and neck squamous cell carcinoma, HPV

## Abstract

Head and neck cancers (HNC) are a biologically diverse set of cancers that are responsible for over 660,000 new diagnoses each year. Current therapies for HNC require a comprehensive, multimodal approach encompassing resection, radiation therapy, and systemic therapy. With an increased understanding of the mechanisms behind HNC, there has been growing interest in more accurate prognostic indicators of disease, effective post-treatment surveillance, and individualized treatments. This chapter will highlight the commonly used and studied biomarkers in head and neck squamous cell carcinoma.

## 1. Introduction

Head and neck cancer (HNC) ranks as the seventh most common cancer worldwide and is responsible for over 660,000 new diagnoses each year [1]. Squamous cell carcinomas, which arise from the epithelial lining of the oral cavity, pharynx, and larynx, comprise approximately 90% of HNCs [1,2,3,4]. The epidemiology of HNC is changing worldwide due to decreasing smoking rates and the rise of HPV-positive tumors, which are primarily of oropharyngeal origin [5,6,7,8]. Despite advances in treatment, there has been a gradual increase in overall mortality, with five-year survival rates of approximately 50–85%, depending on the type and location of the tumor [1,5,6]. Treatment of HNC confers significant morbidity and impacts the quality of life [9,10,11,12].

Current therapies for HNC require a multimodal approach that may include surgical resection, radiation therapy, and/or systemic therapy [2,9,13]. With the growing understanding of the mechanisms behind HNC, there has been interest in more accurate prognostic indicators and effective post-treatment surveillance, as well as alternative individualized treatments. In this chapter, we review the existing literature regarding molecular biomarkers for various types of HNC.

This article reviews common diagnostic, prognostic, and predictive biomarkers, with an emphasis on viral-mediated vs. non-viral mediated disease. The role of human papillomavirus (HPV) has been increasingly recognized in the pathophysiology of oropharyngeal squamous cell carcinoma (OPSCC), such that two distinct disease entities are now recognized: HPV-negative disease associated with risk factors such as tobacco and alcohol use, and HPV-positive disease [2,14]. In addition to HPV, the Epstein–Barr virus (EBV) has also been recognized as playing a role in various head and neck cancers, most notably nasopharyngeal carcinoma [15,16].

Diagnostic biomarkers can confirm the presence of a disease or identify patients with subtypes of a disease [17]. Prognostic biomarkers predict a cancer’s outcomes irrespective of any specific treatment administered and reflect the intrinsic aggressiveness of the malignancy. In contrast to predictive biomarkers, the presence of a prognostic biomarker does not affect the treatment benefit [18]. Predictive biomarkers predict the outcome of a particular treatment, often through a comparison between two treatment approaches [18]. These biomarkers may be especially relevant when designing clinical trials, as they offer insights into identifying patient cohorts that may benefit most from a treatment [19]. There is also overlap between prognostic and predictive biomarkers, so the relevant biomarkers in these categories have been combined. A list of the mentioned biomarkers is available in Table 1.

## 2. HPV-Driven HNCs

Human papillomavirus (HPV) is a primary cause for most (70–90%) OPSCCs and some non-OPSCCs in the United States [20,21]. HPV-positive disease has been shown to have a more favorable response to treatment and improved survival when compared to HPV-negative disease [2,22]. HPV16 accounts for most cases [2]. Methods for discerning HPV-driven oncogenesis in tumor tissue are varied, with emerging evidence that the prognosis differs according to how HPV-positivity is defined. In addition, several HPV-specific minimally invasive biomarkers have recently been identified, leading to an increased interest in early diagnosis and screening [2].

### 2.1. Direct vs. Indirect HPV Testing in Tumor Tissue

HNCs may be evaluated for the presence of HPV-mediated disease via direct methods, e.g., the detection of HPV DNA or mRNA expressed in tumor tissues, or via indirect methods, e.g., the identification of p16^Ink4a^ (p16) expression via immunohistochemistry.

P16 is a known biomarker of HPV oncogenesis that is considered an indirect marker of HPV-positive disease in OPSCC [23]. It is a tumor-suppressor protein and cell-cycle regulator that is overexpressed as a result of HPV-driven oncogenesis and detected via immunohistochemical evaluation, whereby at least 70% nuclear and cytoplasmic expression is considered p16-positive. Importantly, p16 is also overexpressed as a result of other, HPV-independent processes and thus is only considered a surrogate for HPV positivity in the oropharynx, where the prevalence of HPV is high and thus the positive predictive value is high. Guidelines by the College of American Pathologists recommend that p16 testing should be performed on oropharyngeal tumor tissue specimens based on previous literature on this biomarker’s utility as an independent predictor of OPSCC prognosis, its widespread availability, and exemplary performance on specimen samples. However, this recommendation only extends to OPSCC, and p16 testing is not recommended for other tumor types including neuroendocrine or salivary gland tumors [24].

Direct methods of HPV detection in tumor tissues include ISH or PCR for HPV DNA or mRNA. The detection of ‘transcriptionally active’ HPV refers to ISH for HPV E6 or E7 mRNA; ISH for HPV DNA is not recommended [24]. These methods are HPV genotype-specific and are less readily available than p16 IHC. Importantly, some evidence suggests that the prognosis for HPV+ OPSCC varies by HPV genotype, suggesting that type-specific methods of HPV detection may be preferred in the future [25].

Up to 20% of patients with p16-positive tumors test negative for HPV DNA or RNA [21]. Other examples of discordant cases exist, where p16-positive patients are HPV-negative and p16-negative patients are HPV-positive. A recent large study of 7654 patients found that discordance between p16 and HPV status negatively impacts prognosis, leading the authors to recommend expanding indications for direct HPV testing [22]. Overexpression of p16 in HPV- tumors appears to happen due to differing mechanisms, necessitating the use of various techniques for detection and the consideration of factors unrelated to HPV status. Mehanna et al. found that when using p16 IHC alone for HPV status determination, approximately 8% of p16-positive patients would be incorrectly classified as having an HPV+ tumor [22]. The authors concluded that while routine HPV and p16 evaluation should be conducted in OPSCC clinical trials and in clinical settings where more accurate counseling is wanted, the classification of patients with OPSCC based on p16 status alone is still inadequate in routine clinical practice [22].

### 2.2. Blood- and Saliva-Based HPV Biomarkers

Antibodies to HPV E6 antigens, especially the E6 protein, are linked to an increased risk of developing oropharyngeal cancer and appear in blood collected up to several decades before the onset of disease [20,26]. At the time of diagnosis, HPV16-E6 seropositivity is a highly sensitive [27,28] and specific [26,27] marker for HPV-positive OPSCC. HPV16-E6 seropositivity is generally stable, and does not have a strong association with the response to treatment or recurrence [20].

Circulating tumor HPV DNA (ctHPVDNA), referring to fragments of DNA shed from tumor cells into the blood, has been identified using highly sensitive PCR or next-generation sequencing techniques in around 90% of patients with HPV-positive OPSCC [29,30]. It is highly specific for malignancy, and is also dynamic, varying with the burden of disease and the response to treatment due to its short half-life, and is under study for use in the post-treatment surveillance of HPV+ OPC [28]. These properties make ctHPVDNA a promising clinical tool that is under study for use in the diagnosis, treatment, and surveillance of HPV-positive OPSCC.

ctHPVDNA has been most extensively studied in the post-treatment surveillance setting, where it has a high (~95–100%) positive and negative predictive value for disease recurrence and may complement PET/CT to improve the accuracy of surveillance compared with the current standard of care [28,31,32,33,34,35,36]. During treatment, dynamic changes in ctHPVDNA are being studied to guide therapy in real-time, including dose adjustment during definitive radiation (e.g., NCT04900623). Among patients treated with surgery, postoperatively detectable ctHPVDNA is correlated with a risk of residual disease and extra-nodal extension, suggesting it is a high-risk feature that warrants consideration of adjuvant therapy [35,37]. In the prediagnostic setting, ctHPVDNA has been detected In blood collected several years prior to the diagnosis of HPV+ OPSCC in a subset of patients [38]. Ongoing research will elucidate whether the incorporation of ctHPVDNA into HPV-positive OPSCC diagnosis, treatment and surveillance paradigms will measurably improve patient outcomes.

Oncogenic HPV DNA found in oral rinses has also been associated with treatment response and recurrence following treatment but has lower sensitivity compared to ctHPVDNA and is subject to more variability [39,40]. Bystander infections or a patient’s inability to clear viral cells following infection are potential confounders, making this method less specific compared to measuring circulating tumor HPV DNA [40,41].

### 2.3. HPV Tumor Status in Non-Oropharynx HNC Sites

HPV is detected in a subset of HNCs outside of the oropharynx (larynx, oral cavity, hypopharynx, nasopharynx); however, the prevalence is much lower than in the oropharynx and the clinical significance is unclear [42]. While the prevalence of HPV positivity has been increasing in laryngeal and oral cavity cancer, there does not seem to be an association between HPV status and survival [43,44]. At this time, testing for HPV in non-oropharynx HNCs is not currently recommended [45]. However, recent research has highlighted the utility of high-risk HPV in understanding the prognosis of sinonasal cancer, with high-risk HPV being associated with a better prognosis in these patients [43,46,47]. Several recent papers have found that the prevalence of HPV positivity in sinonasal cancer has been increasing, with favorable survival being associated with HPV status [48,49].

## 3. Prognostic and Predictive Molecular Markers in HPV-Positive Tumors

### 3.1. HPV Viral Integration

A more recent area of focus in HPV-positive head and neck cancer is HPV viral integration into the host cellular genome. Viral integration is associated with higher transcription rates of E6 and E7 and the progression of carcinoma in cervical cancers [50,51], and has been appreciated in head and neck carcinoma. However, the association between viral integration and clinical outcomes is not well understood [52], and current evidence is often conflicting, with variability in methods for detecting viral integration. While there is some evidence for the epigenetic upregulation of regions around the integrated HPV genome in the host, the clinical significance of these findings is under investigation [53].

### 3.2. HPV Subtype

The HPV genotype is not a widely used measure of risk stratification for head and neck squamous cell carcinoma. This is largely due to the relative homogeneity of the HPV genotype in North America, with only around 8–14% of oropharyngeal cancers having genotypes other than HPV-16 [54,55]. However, emerging literature suggests that tumors associated with HPV-16 tend to have better prognoses compared to other genotypes, with a five-year overall survival of 83% versus 69% in HPV16 and HPV-non16, respectively, in a recent meta-analysis of 1310 HPV16 and 219 HPV-non16 patients [25,56,57,58]. Further studies should be carried out to examine the HPV genotype and its role in prognosis and treatment decision-making.

### 3.3. Estrogen Receptor Positivity

Recent studies have examined tumor estrogen receptor alpha (ERa) positivity as a biomarker for improved overall survival and recurrence-free survival in HPV-positive oropharyngeal cancer [52,53]. While ERa is a biomarker and therapeutic target for breast cancer, its utility in head and neck squamous cell carcinoma has only recently been investigated. Several studies have found an improved prognosis in patients with Era-positive oropharyngeal squamous cell carcinoma treated with chemoradiation, even after accounting for various clinical risk factors [59,60,61]. While there are no current studies investigating ERa as a therapeutic target in head and neck cancer, its presence in tumors may help guide treatment selection and de-intensification [59].

### 3.4. Apolipoprotein B mRNA Editing Enzyme, Catalytic Polypeptide

The apolipoprotein B mRNA editing enzyme catalytic polypeptide-like (APOBEC) family of cytidine deaminases is made up of aberrantly-acting DNA-modifying enzymes that have been linked to DNA mutations and tumor formation [62,63]. APOBEC activation has a role in the immune system’s response to viruses including HPV and EBV [64,65]. Recent studies have shown higher APOBEC activity in HPV-positive HNC [66,67], as well as a correlation between the mutational burden of HPV-positive head and neck cancer with APOBEC enrichment [14,68,69]. Patients with HNC have an improved treatment response to chemotherapy and immunotherapy, with a higher expression of certain A3H or A3G proteins being correlated with increased survival [67,69,70,71].

## 4. Role of EBV in Nasopharyngeal Cancer

The Epstein–Barr virus (EBV) is closely linked to the pathogenesis of nasopharyngeal cancer, a distinct form of HNC endemic to the Asian population. EBV is a ubiquitous virus that infects a majority of the global population, usually establishing a lifelong latent infection in B lymphocytes. In the context of NPC, EBV plays a pivotal role in oncogenesis by promoting genetic alterations, immune evasion, and the dysregulation of cellular signaling pathways [72,73]. The complex interplay of viral and host factors involved in EBV-associated nasopharyngeal carcinoma allows for the use of diagnostic, prognostic, and predictive biomarkers.

## 5. Biomarkers for the Screening and Diagnosis of EBV-Positive Nasopharyngeal Cancer

Serologic testing for antibodies to EBV antigens has been used to evaluate patients with suspected nasopharyngeal carcinoma, and for the screening of endemic populations [73,74]. In particular, the EBNA1 peptide has been shown to have optimal performance characteristics when detecting asymptomatic nasopharyngeal carcinoma. Using optimized threshold values, the sensitivity of EBNA1 IgA has been shown to be 80–85.7% [73,74], with a specificity of 51.2% in one study [73]. A novel biomarker, anti-BNLF2b total antibody (P85Ab), is a promising biomarker found to have a higher sensitivity, specificity, and positive predictive value compared to the standard two-antibody-based screening method (EBV nuclear antigen 1 IgA and EBV viral capsid antigen IgA). The positive predictive value was also increased, which may improve the cost-effectiveness of screening [75]. While promising, further prospective studies and randomized controlled trials are necessary to further guide the use of these biomarkers [76].

The detection of cancer-related EBV DNA in the bloodstream has been confirmed as a reliable indicator for nasopharyngeal carcinoma [72]. EBV DNA has been detected in 96% of patients with nasopharyngeal carcinoma using quantitative real-time polymerase chain reaction and has shown utility in determining the prognosis and detecting residual disease [77,78]. Several studies have also examined the utility of using EBV DNA in plasma to screen for asymptomatic cases of nasopharyngeal carcinoma at an early disease stage, with promising results [72,79,80,81]. In a prospective study of asymptomatic patients, a screening test that marked patients as positive if they had tested positive for EBV DNA twice, four weeks apart, showed a greater than 97% sensitivity, specificity, and negative predictive value. Of those with detected nasopharyngeal carcinoma, 70% had stage 1 or 2 disease, which is significantly higher than historical cohorts that showed a greater percentage of late-stage disease [74].

## 6. Prognostic and Predictive Molecular Markers in EBV Positive Nasopharyngeal Cancer

Patients with nasopharyngeal carcinoma have been shown to have high serologic titers against viral antigens, as discussed above. Recent studies have focused on circulating EBV DNA as a non-invasive and clinically useful biomarker for the prognosis of EBV, with studies showing a higher risk of mortality, recurrence, and metastasis associated with higher levels of pre-treatment EBV DNA compared to those with low levels [82,83,84]. However, many of these studies are limited to single-institution studies or smaller cohorts, with little variation in ethnic variety that can influence prognosis. Furthermore, patient management was not standardized between studies, which may influence the results.

## 7. Prognostic and Predictive Molecular Markers in Virus-Negative HNCs

Many HNCs are not driven by viral oncogenesis, but rather chemical carcinogens, most commonly tobacco [85]. The clinical and molecular profiles of these tumors are distinct from those of virally driven cancers, and they are considered to be separate disease entities. Clinically, HNCs that are not associated with HPV or EBV occur in all subsites of the head and neck, most commonly in middle-aged and older men who smoke. While the incidence of this form of HNC is decreasing, it tends to have a poorer prognosis. There are a number of biomarkers that are utilized in virus-negative tumors with diagnostic, prognostic, and predictive significance. These are presented beginning with those that are currently used in clinical practice followed by those that are still under investigation.

### 7.1. TP53 and P53

The expression of the tumor-suppressor protein p53 is altered by mutations in the TP53 gene, which are prevalent in various cancers [85]. Previous studies have found that aberrant forms of p53 proteins, in conjunction with TP53 mutations, can confer increased resistance of HNC to chemotherapy [86,87]. The p53 tumor-suppressor protein has oncoprotective functions, including the modulation of reactive oxygen species, the regulation of the G1/S phase of the cell cycle, and the induction of apoptosis [86,88]. Levels of p53 overexpression in HNC vary due to differing risk factors and pathogenesis across the world [89,90,91]. The loss of p53 expression or the overexpression of a mutant p53 protein are associated with a poor prognosis, increased extranodal extension, higher tumor grades, and a higher rate of recurrence in HNC [88,92]. Treatment strategies to restore the wild-type function of p53 or induce the degradation of mutant p53 proteins are challenging because they require knowledge of a specific p53 mutation to be effective [87].

### 7.2. EGFR

Epidermal growth factor receptor (EGFR) is overexpressed in a variety of solid tumors, including approximately 80% of HNC [93]. Heightened EGFR expression is associated with a poorer prognosis and a more aggressive tumor presentation [93,94,95]. Multiple treatment approaches targeting EGFR have been explored, including the extracellular domain, intracellular domain, and at the genetic level [96]. For example, EGFR-specific monoclonal antibodies bind to the receptor and block ligand binding, resulting in the blockade of EGFR phosphorylation and downstream signaling. The use of cetuximab in combination with radiotherapy in patients with locoregionally advanced HNC demonstrated an improvement in the duration of locoregional control [97]. Various combinations have been attempted, including the use of cetuximab as a monotherapy [98] or in combination with cisplatin and radiotherapy [99]. Tyrosine kinase inhibitors are another class of drugs that blocks tyrosine kinase enzymes, thus reducing downstream signaling in this pathway [100]. Various treatment methods and combinations with chemo- and radiotherapy have been attempted, similarly to EGFR-specific monoclonal antibodies [100].

### 7.3. PD-L1 Expression

Programmed death-ligand 1 (PD-L1) is a pivotal immune checkpoint regulator expressed in various immune cells [101]. In HNC, the interaction of PD-L1 and its receptor, programmed cell death protein 1 (PD-1), leads to the suppression of immune responses, and particularly the inhibition of T cell activation. This interaction effectively hampers the body’s natural ability to recognize and eliminate cancer cells. The upregulation of PD-L1 in the tumor microenvironment creates an immunosuppressive shield, allowing cancer cells to evade detection and destruction by the immune system. Consequently, this molecular interplay promotes tumor progression [102].

In the context of therapeutic interventions, efforts have been directed toward disrupting the PD-1/PD-L1 axis to restore effective antitumor immune responses. Anti-PD-1 antibodies, such as nivolumab and pembrolizumab, block the interaction between PD-L1 and PD-1, thereby unleashing the immune system to mount a more robust attack against cancer cells. This targeted immunotherapy demonstrated improved survival outcomes for patients with HNC [103,104]. Consequently, immunotherapy now plays a central role in the treatment of many HNCs, marking a significant shift in the treatment landscape.

However, the nuanced role of PD-L1 in HNC treatment extends beyond being a therapeutic target. Other studies have examined the prognostic value of PD-L1, and have found it to be an independent risk factor for oral squamous cell carcinoma, with increased expression being associated with distant metastasis and poor overall survival [105,106]. However, other studies found that the increased expression of PD-L1 was associated with longer disease-free survival in high-risk head and neck squamous cell carcinoma [107]. Limitations to the use of PD-L1 include its unknown significance in head and neck squamous cell carcinoma apart from oral cavity cancer, and the lack of data on the PD-L1 ligand in head and neck squamous cell carcinoma [108].

### 7.4. Beta 2-Microglobulin

β2-microglobulin (β2M) is a component of the major histocompatibility complex (MHC) class I and holds significance in assessing tumor status in various cancers [109,110,111]. While β2M is typically present at low physiological levels, elevated levels have been observed in conditions like renal failure and certain malignancies, including oral SCC. This suggests that elevated β2M could serve as an effective diagnostic marker [112]. Levels may be assessed in the blood as well as saliva, making fast and cost-effective screening possible [113,114]. β2M levels have been studied not only for diagnostic purposes but also as a potential marker for tumor progression, metastasis, and survival in patients with oral cavity SCC [110,115]. Most studies examining β2M are limited by small sample sizes, and β2M has not been sufficiently validated for use as a biomarker in various clinical settings [113].

### 7.5. Hypoxia Markers

Hypoxia, defined as a mismatch between oxygen supply and demand [116] has been studied in the context of solid tumors including head and neck squamous cell carcinoma [117]. Tumor growth intensifies the requirement for sufficient cellular oxygenation, which often exacerbates hypoxia [118]. Hypoxia is linked to tumor progression, as it modifies the free radical chemistry of tumors and contributes to a more aggressive phenotype. As a result, there is a decreased sensitivity to radiotherapy and some forms of chemotherapy [116,119]. In addition, hypoxia can lead to increased genetic instability, mutations, an antitumor immune response, and the evolution of genetically hypoxia-resistant phenotypes [111,120,121].

The most important endogenous biomarkers for hypoxia include the hypoxia-inducible factor (HIF)-1a and -2a pathways, glucose transporter (GLUT)-1, CA-IX, and osteopontin (OPN), among others. In a systematic review, the expression of endogenous hypoxia biomarkers was commonly observed and associated with poorer survival and locoregional control in most studies, along with unfavorable clinicopathological tumor characteristics [116].

### 7.6. Neutrophil to Lymphocyte Ratio

Chronic inflammation is implicated in tumorigenesis across various cancers, including HNC [122,123,124]. The neutrophil-to-lymphocyte ratio (NLR) characterizes the inflammatory response to cancer and has emerged as predictive of outcomes in solid tumors [125]. Studies consistently demonstrate that a high pre-treatment NLR is associated with poor overall and progression-free survival as well as an increased likelihood of tumor recurrence [126,127]. Interestingly, a recent study showed that a very low pre-NLR seemed to have a similar effect on prognosis [128]. Post-treatment NLR has also been utilized in a similar fashion to pre-treatment values [122,129]. Interestingly, a recent study showed that a very low pre-operative NLR may yield a similar negative effect on prognosis [105]. Given the easy availability, objectivity, and cost-effectiveness of NLR, it may be advantageous to other biomarkers [128].

### 7.7. PTEN

PTEN is a tumor suppressor that negatively regulates the PI3K–AKT–mTOR pathway and various processes related to cell growth and proliferation [130]. PTEN mutations have been detected in numerous tumor types, including breast, blood/lymph, central nervous system, and thyroid, among others [131,132]. Assessments of the effect of PTEN expression on the prognosis of HNC yield mixed results, with some demonstrating that increased PTEN expression is associated with a favorable outcome following treatment [130,133], while others suggest that heightened expression is linked to poor outcomes [134]. The current literature highlights the challenge of making robust comparisons between groups due to the diverse methodologies employed for IHC scoring of PTEN.

### 7.8. Cyclin D1

The G1 checkpoint, a crucial determinant governing the progression of cell division [62], is tightly regulated by a group of proteins including cyclins, cyclin-dependent kinases (CDKs), and CDK inhibitors [135]. Cyclin D1 has been implicated in the development of tumors of the esophagus, ovary, breast, colon, lung, and head and neck [136]. Previous studies have established associations between the overexpression of cyclin D1 and the development of regional lymph node metastases in HNC [137,138,139], as well as decreased disease-free and overall survival [104,140,141,142].

### 7.9. ERCC1

Excision repair cross-complementation group 1 (ERCC1) plays an important role in the nucleotide excision repair pathway of DNA repair [143]. ERCC1 gene polymorphisms have been associated with an increased risk of certain cancers, including nasopharyngeal carcinoma [144], lung cancer [145], melanoma [146], and pancreatic cancer [147]. Increased expressions of ERCC1 and related polymorphisms are associated with decreased progression-free survival [148], increased susceptibility to nasopharyngeal carcinoma [144], increased rates of recurrence in patients treated with concurrent chemoradiotherapy [149], and increased resistance to radiotherapy [150]. These studies highlight a possible role for more aggressive treatment in these patients and may allow clinicians to predict which patients might benefit from platinum-based chemotherapy [151].

### 7.10. Cathepsin D

Cathepsin D is a lysosomal enzyme found throughout the body’s cells. It has been found to be overexpressed and/or abnormally processed in various cancer cells and is thought to have a role in local tumor invasion and metastasis [152]. While cathepsin D has been studied mostly in the context of breast cancer patients, several authors have examined its role in head and neck squamous cell carcinoma [152,153,154,155]. Several studies have found that cathepsin D gene expression was associated with metastasis in head and neck squamous cell carcinoma [154,155,156].

### 7.11. Bcl-2

Bcl-2 is a key mitochondrial protein that regulates apoptosis, with overexpression of the protein neutralizing pro-apoptotic proteins and inhibiting apoptosis [157]. In HNC, the role of Bcl-2 as a prognostic and predictive biomarker remains equivocal, with conflicting evidence. Several studies have found Bcl-2 overexpression to be an independent risk factor for HNC [158,159], as well as an independent poor prognostic factor [157,160]. However, others have contrarily reported that a negative Bcl-2 expression correlates with a worse prognosis [161,162] or indicated no association between Bcl-2 expression and prognosis or tumor aggressiveness [162,163,164].

### 7.12. Tumor Budding and Epithelial–Mesenchymal Transition

Tumor budding, defined as the presence of single tumor cells or groups of tumor cells at the tumor margin [165], has been established as a prognostic factor in colorectal cancer, pancreatic cancer, esophageal cancer, and breast cancer [166,167,168]. In HNCs, tumor budding has been described as a prognostic factor for lymph node metastasis and early-stage OPSCC [169,170]. Tumor budding has also been studied in association with epithelial–mesenchymal transition, which involves the transition of an epithelial cell to a mesenchymal cell phenotype [171]. This transition, thought by some to play a role in the initiation of tumor budding, is associated with the conversion of cancer cells into a more invasive and metastatic phenotype. This enhanced cellular state enables cancer migration to other regions of the body. Tumor budding cells have been found to express various molecules such as ZEB1, ZEB2, E-cadherin, and SNA1, which are characteristic of epithelial–mesenchymal transition [167,172]. Some studies suggest that tumor budding and epithelial–mesenchymal transition are independent processes in OPSCC progression [173], and others have found significant associations between the two processes [174,175].

### 7.13. DNA Methylation

Known contributors to OPSCC such as tobacco, alcohol abuse, and HPV positivity confer increased risk through epigenetic changes such as DNA methylation [176,177,178]. DNA methylation occurs through the addition of a methyl group to a carbon of cytosine, forming 5-methylcytosine [177]. This change in DNA structure leads to a change in the activity of transcription factors and the mobility of various proteins [179]. Tobacco and alcohol use have been associated with hypomethylation in HNC, while HPV-positive tumors have been associated with hypermethylation [179]. Furthermore, differing degrees of methylation have been linked to different tumor locations, and various sites of methylation have been shown to be correlated with more aggressive disease progression [177,180,181]. There is a need for further studies on this topic, given the rapidly expanding identification of new biomarkers and the need to transfer this knowledge to clinical practice.

### 7.14. MicroRNAs

MicroRNAs (miRNA), molecules of non-coding DNA that regulate gene expression and dysregulation [182], have been associated with the initiation and progression of malignancy [183]. Studies have shown them to be prognostic biomarkers in B-cell lymphomas [184], lung cancers [185], and hepatocellular carcinoma [186]. This is a biomarker of interest in HNC because the miRNA expression profiles have been found to differ between HPV-positive and -negative HNCs [187,188]. Different miRNA signatures have been found to improve the risk stratification of HPV-positive and -negative tumors [189,190]. One study of HPV-negative HNC patients who received chemoradiotherapy reported that a five-miRNA signature (hsa-let-7g-3p, hsa-miR-6508-5p, hsa-miR-210-5p, hsa-miR-4306, and hsa-miR-7161-3p) was a strong prognostic indictor for recurrence and survival. Other studies are also investigating its utility in the evaluation of treatment response in HNCs [189,191].

## 8. Emerging Biomarkers

Several other emerging biomarkers have been studied in recent decades regarding their role as prognostic biomarkers in HNC. These include beta tubulin isotypes, the proteasomal degradation protein PSMD14, the T cell regulator SSP1, and the matrix metalloproteinase (MMP) family of enzymes that degrade the extracellular matrix [192,193,194,195]. Beta tubulin II and III have been implicated in predicting outcomes of taxane and cisplatin-based chemotherapy in HNC with promising results [151,193,196]. However, their utility in a clinical setting has yet to be established and further research needs to be conducted. PSMD14 is another protein that is associated with poor progression and an advanced tumor stage [194,197], with some research being conducted on its potential role as a therapeutic target in the Akt pathway [197]. SSP1 has been studied in terms of its ability to modulate the immunosuppressive mechanisms of tumor cells, especially in patients treated with Nivolumab [195]. The MMP family of enzymes has also been shown to be involved in immune cell infiltration in HNC, specifically MMP14, MMP16, and MMP19 [198]. Future studies should be conducted to standardize the measurement of these biomarkers, verify their expression levels, and further establish their clinical significance.

## 9. Conclusions

Biomarkers provide an opportunity to advance head and neck cancer care by potentially leading to earlier diagnosis, improving prognostication, aiding treatment decision making, and helping identify recurrences post-treatment. Several such biomarkers are now routinely utilized in HNC care. These include p16, HPV, and ctHPVDNA in the HPV-positive population; EBV and anti-EBV antibodies in the EBV-positive population; and p53, EGFR, and PDL1 in the non-virally induced cancer population; with a myriad of other biomarkers being studied.

As medicine evolves to become more individualized, there arises a heightened demand for reliable and clinically applicable biomarkers to contribute to the effective management of patients suffering from HNC.

## Figures and Tables

**Table 1:** List of biomarkers mentioned in this review.

Biomarker	Description
Diagnosis	
EBV status	Serologic tests examining antibodies to EBV IgA are performed to evaluate patients with suspected nasopharyngeal carcinoma.
Beta 2-microglobulin	A component of the major histocompatibility complex (MHC) class I. It is associated with tumor status in various cancers.
Diagnostic/Prognostic	
p16^Ink4a^	An indirect marker of HPV-positive disease in oropharyngeal squamous cell carcinomas.
HPV-E6 seropositivity	Antibodies to the HPV virus E6 antigen. Linked to an increased risk of developing oropharyngeal cancer.
Circulating tumor HPV DNA	Fragments of HPV DNA shed from tumor cells into the blood; highly specific for malignancy.
Oral HPV DNA	Oncogenic HPV DNA found in oral rinses. Has been associated with treatment response and recurrence following treatment but has been shown to have less sensitivity compared to ctHPVDNA.
Prognostic	
Estrogen receptor positivity	Tumor estrogen receptor alpha (Era) positivity, being studied as a biomarker for improved overall survival and recurrence-free survival in HPV-positive oropharyngeal cancer.
Hypoxia markers	Hypoxia is associated with tumor progression, contributing to a more aggressive phenotype and modifying the free radical chemistry of tumors.
TP53 and P53	A tumor-suppressor gene that regulates gene overexpression in head and neck squamous cell carcinoma.
Cyclin D1	Regulator of the G1 checkpoint of cell division. D1 has been implicated in the development of tumors of the esophagus, ovary, breast, colon, lung, and the head and neck.
Cathepsin-D	A lysosomal enzyme found throughout the body’s cells. It has been found to be overexpressed and/or abnormally processed in various cancer cells and is thought to have a role in local tumor invasion and metastasis.
Bcl-2	A key mitochondrial protein that regulates apoptosis, with overexpression of the protein neutralizing pro-apoptotic proteins and inhibiting apoptosis in the cell.
Prognostic/predictive	
Apolipoprotein B mRNA Editing Enzyme, Catalytic Polypeptide (APOBEC)	Aberrantly acting DNA-modifying enzymes that have been linked to DNA mutations and tumor formation.
Neutrophil to Lymphocyte Ratio	The balance of neutrophils and lymphocytes, expressed as a neutrophil-to-lymphocyte ratio (NLR), has been shown to predict outcomes in solid tumors.
PTEN	A tumor suppressor that negatively regulates the PI3K–AKT–mTOR pathway and regulates various processes related to cell growth and proliferation.
ERCC1	Excision repair cross-complementation group 1 (ERCC1) is an important part of the nucleotide excision repair pathway of DNA repair.
PD-L1 Expression	PD-L1 is an immune checkpoint regulator and immunomodulatory protein expressed in various immune cells.
EGFR	Epidermal growth factor receptor, overexpressed in a variety of solid tumors, with significant representation in head and neck squamous cell carcinoma.
Tumor budding and epithelial–mesenchymal transition	Tumor budding is defined as the presence of single tumor cells or groups of tumor cells at the tumor margin. Epithelial–mesenchymal transition involves the transformation of an epithelial cell into a mesenchymal cell phenotype.
DNA methylation	DNA methylation occurs through the addition of a methyl group to a carbon of cytosine, forming 5-methylcytosine. This leads to a change in the activity of transcription factors and the mobility of various proteins.
MicroRNAs	MicroRNAs are molecules of non-coding DNA that regulate gene expression and dysregulation.
Beta-tubulin isotypes	Beta-tubulin isotypes regulate the structure of microtubules.
PSMD14	PSMD14 is a proteasomal degradation protein that removes ubiquitin from proteins.
SSP1	SSP1 prevents the proliferation of effector T cells.
Matrix metalloproteinase family of enzymes	Matrix metalloproteinases participate in a variety of biological processes including the degradation of tissue components, angiogenesis, and neurogenesis.

## Data Availability

No new data were created or analyzed in this study. Data sharing is not applicable to this article.
